# Endotoxin, dust and exhaled nitrogen oxide among hand pickers of coffee; a cross-sectional study

**DOI:** 10.1186/s12995-016-0108-7

**Published:** 2016-04-12

**Authors:** Bente Elisabeth Moen, Akwilina Kayumba, Gloria Sakwari, Simon Henry David Mamuya, Magne Bråtveit

**Affiliations:** Department of Global Public Health and Primary Care, Centre for International Health, University of Bergen, Postbox 7804, NO-5020 Bergen, Norway; Occupational Safety and Health Authority, Dar es Salaam, Tanzania; Muhimbili University of Health and Allied Sciences, Dar es Salaam, Tanzania; Department of Global Public Health and Primary Care, University of Bergen, Bergen, Norway

**Keywords:** Coffee, Coffee bean processing, Dust, Endotoxin, Exhaled nitric oxide, Hand picking, Respiratory inflammation

## Abstract

**Background:**

Primary coffee processing takes place in countries where coffee is grown, and may include hand picking of coffee to remove low quality beans. Hand picking is mostly performed by women. No previous studies on dust and respiratory health have been performed in this occupational group, although studies indicate respiratory problems among other coffee production workers.

**Findings:**

Our aim was to assess dust and endotoxin exposure among hand pickers in a coffee factory and compare the levels with limit values. In addition we wanted to examine the fraction of exhaled nitric oxide (FeNO) as a possible inflammatory marker in the airways among the hand pickers and evaluate the association between FeNO and years of hand picking. All hand pickers in a factory were examined during 1 week. The response was 100 %; 69 participated. FeNO was measured using an electrochemistry-based NIOX MINO device. Nine out of 69 workers (13 %) had levels of FeNO above 25 ppb, indicating presence of respiratory inflammation. A significant positive association was found between increasing FeNO and years of hand picking. Nine personal samples of total dust and endotoxin were taken. None of the dust samples exceeded the occupational limit value for total organic dust of 5 mg/m^3^. Three samples of endotoxin (33 %) were above the recommended value of 90 EU/m^3^.

**Conclusions:**

Levels of endotoxin were higher than recommended standards among hand pickers, and there was a positive association between the level of exhaled nitrogen oxide and years of work with hand picking coffee.

## Background

Several studies have indicated that dust exposure in coffee factories may cause respiratory problems among workers [1–3]. Several of the studies are quite old [4, 5] and have been performed in coffee importing factories [1, 4, 5]. However, respiratory problems related to coffee may also affect workers performing primary coffee processing in exporting countries [2], which has also been shown in relatively recent studies [3]. A possible relationship between coffee dust exposure and airway obstruction during primary coffee processing work has been shown [6]. It has been suggested that endotoxin in the coffee dust might be a causative agent related to these respiratory problems [6, 7].

In the coffee producing countries, coffee is delivered from farms to factories for primary processing; cleaning, hulling, and grading, before export. These work tasks are mainly performed by men. However, primary coffee factories may also provide an extra quality check of the coffee beans, called “hand picking”. This process is performed by women only; they remove low quality, discoloured beans by hand. The women in the factory included in this study, sat at high tables and sorted the beans. The beans were provided to them in bags, and they picked up beans from the bag using a small box. The women picked out the low quality beans using their hands and pushed the final, perfect beans into a new coffee bag when finished. They all performed the same task in the same manner and were sitting in the same room under very similar working conditions. To our knowledge, no previous studies of dust or respiratory health have been performed in this occupational group.

The aim of this study was to assess dust and endotoxin exposure among hand pickers in a coffee factory and compare the dust levels with occupational limit values. Total dust was assessed as some countries have limit values for this parameter. Endotoxin level was assessed since it has been suggested that this component of organic dust may cause adverse respiratory health effects [8]. In addition we wanted to examine the fraction of exhaled nitric oxide (FeNO) among the hand picking workers, as an objective indicator of eosinophilic inflammatory reactions in the airways [9]. A coffee bean allergen has been isolated [10] and it might be possible for this type of workers to develop allergies [5], thus examination of FeNO was chosen for the study. FeNO is a biomarker of airway inflammation in different populations [11, 12]. It has in particular been described as a marker of lower airway Th2 driven (allergic) inflammation [13]. We also wanted to evaluate the association between levels of FeNO and years of hand picking work. Knowledge about hand picking work and health is important for implementation of correct preventive measures in this occupational group.

## Methods

A cross sectional study was performed among all hand picking workers in the production of a primary coffee producing factory in Tanzania during 1 week, in November 2009. The factory had in total 117 persons employed, including 72 female hand pickers. All hand pickers were asked for participation by the researchers, and everyone agreed to participate and gave their written informed consent.

The participants were interviewed about age, years worked with hand picking coffee and background variables for possible exclusion. Exclusion criteria were: Daily smoking, use of anti-inflammatory medication and any other medication for respiratory or cardiovascular diseases, previous tuberculosis, pneumonia or HIV and present fever or a cold [9, 13]. Three women were excluded; two were daily smokers and one had a fever at the time of examination, and the final number of participants was 69.

The participants were examined during day shift by measuring FeNO, using an electrochemistry-based NIOX MINO device (Aerocrine AB, Solna, Sweden), fitted with a NIOX MINO 300 sensor pre-calibrated from the manufacturer in 2009. The measurements were performed in accordance with the ATS/ERS guidelines [14, 15]. The ambient air temperature varied between 24 and 32 °C, humidity was 60–62 % and NO in ambient air was less than five ppb during measurements.

All hand picking women worked in one room, and ten of them were randomly selected for dust sampling. One sample was lost due to technical problems. Personal dust sampling was conducted by using Sidekick Casella pumps (2.0 l/min; mean sampling time 414 min) connected to 37 mm Millipore closed-faced filter cassettes with polycarbonate filters of 0.8 μm pore size. The samples were stored cold until analysis at the Section of Medical Microbiology, Lund University, Sweden.

Total dust levels were evaluated by gravimetric analysis. Endotoxins extracts were analysed by kinetic chromogenic Limulus Amoebocyte Lysate (LAL) Assay in a similar procedure as described by Douwes et al. [16]. Two filter blanks were included.

E-log transformed values of FeNO were used in multiple linear regression analyses evaluating the association between FeNO and years of hand picking among the workers, while adjusting for age. Years at work and age were significantly correlated (Pearson correlation coefficient 0.6) and therefore years at work were not included in the statistical analysis. PASW statistics version 21 was used; significance level was set to 0.05.

The Regional Committee on Medical Research Ethics, Western Norway (2009/1947) and the National Institute for Medical Research in Tanzania approved the study.

## Results

None of the dust samples exceeded the occupational limit value for total organic dust of 5 mg/m^3^, used in Norway (Table 1). Three samples of endotoxin were above Dutch recommended values of 90 EU/m^3^ [17].

**Table 1:** Total dust and endotoxin level during a normal day of hand picking coffee at a coffee factory; nine samples were taken (3 days of sampling), mean sampling time was a full work day for a hand picker, in our study 414 min (range 390–430)

	Geometric mean (geometric standard deviation)	Minimum-maximum
Total dust (mg/m^3^)	0.9 (0.5)	0.3–1.7
Endotoxin (EU/m^3^)	183 (119)	29–372

The mean level of FeNO was 17 ppb (Table 2). Nine workers had levels of FeNO exceeding 25 ppb. There was a significant positive relationship between FeNO (e-log transformed values) and years of hand picking work in coffee production, using a linear regression analysis including age in the model (β = 1.2, 95 % confidence interval 0.4–2.4, *p* = 0.04).

**Table 2:** Fractional exhaled nitrogen oxide (FeNO), age and years worked with hand picking in primary coffee production among 69 hand pickers

	Mean (standard deviation)	Median	Minimum-maximum
Age	31 (10)	34	19–66
Years worked with hand picking coffee	4 (4)	4	1–17
FeNO (ppb)	17 (18)^a^	13	3–107^b^

^a^Geometric mean:12 and geometric standard deviation: 12. ^b^Two outliers; values 86 and 107

## Discussion

Endotoxin levels in the air of the hand pickers were above recommended limit values in three of nine samples. Nine out of 72 workers had levels of FeNO above 25 ppb, indicating presence of low levels of respiratory inflammation [18]. A positive association between increasing FeNO and years of hand picking in coffee production was found.

The findings are in line with findings from previous studies among other production workers in primary coffee processing, showing a possible relationship between coffee dust exposure and adverse respiratory health [2, 5–7]. The presently measured exposure data show the presence of endotoxin levels above limit values, and may indicate that “years of hand picking” corresponds to years of high endotoxin exposure. The endotoxin exposure might be related to the signs of respiratory inflammation among the workers. However, “Years of hand picking” is a crude indicator for dust exposure, and other unknown exposure factors not measured may also have influenced the levels of FeNO.

The cross-sectional design of the study is a weakness. This is a very small cross-sectional study without a control group. Future studies should be longitudinal to be able to examine the possible causal relationship between the hand picking work and respiratory health problems. Future studies should also include a larger population and examine them by other methods than FeNO. However, a strength of the present study is that regression analyses and scientifically sound measurement methods with calibrated instruments and standard, validated procedures were used. Possible confounding factors like medication, smoking and other diseases were controlled by exclusion criteria and age was controlled by statistical adjustments. The response was 100 % among the workers. It is therefore possible that the relationship found between FeNO and work in coffee might be valid, although other studies of this occupational group are needed to confirm this conclusion.

It seems of importance to include hand pickers in dust reduction programs in primary coffee factories and also to include them in future studies of health in these factories.

## References

[1] Oldenburg M, Bittner C, Baur X (2009). Health risks due to coffee dust. Chest.

[2] Sekimpi DK, Agaba EF, Okot-Nwang M, Ogaram DA (1996). Occupational coffee dust allergies in Uganda. Afr Newsl Occup Health Safety.

[3] Sakwari G, Bråtveit M, Mamuya SHD, Moen BE (2011). Dust exposure and chronic respiratory symptoms among coffee curing workers in Kilimanjaro: a cross sectional study. BMC Pulm Med.

[4] Zuskin E, Valic F, Skuric Z (1979). Respiratory-Function in Coffee Workers. Br J Ind Med.

[5] Osterman K, Zetterstrom O, Johansson SGO (1982). Coffee Worker’s Allergy. Allergy.

[6] Sakwari G, Bråtveit M, Mamuya SHD, Moen BE (2013). Respiratory Symptoms, fractional exhaled NO and lung function among workers in Robusta and Arabica coffee factories. J Occup Environ Med.

[7] Moen BE, Sakwari G, Mamuya SH, Akwilina AV, Larsson L, Pehrson C, Mashalla YJ, Bråtveit M (2012). Respiratory Inflammation Among Workers Exposed to Airborne Dust With Endotoxins in a Coffee Curing Factory. J Occup Environ Med.

[8] Liebers V, Raulf-Heimsoth M, Bruning T (2008). Health effects due to endotoxin inhalation (review). Arch Toxicol.

[9] Sandrini A, Taylor DR, Thomas PS, Yates DH (2010). Fractional exhaled nitric oxide in asthma: an update. Respirology.

[10] Manavski N, Peters U, Brettschneider R, Oldenburg M, Baur X, Bittner C (2012). Cof a 1: identification, expression and immunoreactivity of the first coffee allergen. Int Arch Allergy Immunol.

[11] Bohadana AB, Hannhart B, Ghezzo H, Teculescu D, Zmirou-Navier D (2011). Exhaled nitric oxide and spirometry in respiratory health surveillance. Occup Med.

[12] Sundblad B-M, Larsson B-M, Palmberg L, Larsson K (2002). Exhaled nitric oxide and bronchial responsiveness in healthy subjects exposed to organic dust. Eur Respir J.

[13] Alving K, Malinovschi A, Horvath I, de Jongste JC (2010). Basic aspects of exhaled nitric oxide. Eur Respire Mon, European Respiratory Society.

[14] Smit LA, Heederik D, Doekes G, Wouters IM (2009). Exhaled nitric oxide in endotoxin-exposed adults: effect modification by smoking and atopy. Occup Environ Med.

[15] American Thoracic Society/European Respiratory Society ( ATS/ERS). Recommendations for standardized procedures for the online and offline measurement of exhaled lower respiratory oxide and nasal nitric oxide 2005. Am J Respir Crit Care Med. 2005;171:912–30. 10.1164/rccm.200406-710ST 15817806

[16] Douwes J, Versloot P, Hollander A, Heederik D, Doekes G (1995). Influence of Various Dust Sampling and Extraction Methods on the Measurement of Airborne endotoxin. Appl Environ Microbiol.

[17] Health Council of the Netherlands. Endotoxins. Health-Based Recommended Occupational Exposure Limit. The Hague: Health Council of the Netherlands 2012: Publication no. 2010/04OSH. ISBN 978-90-5549-8.

[18] Stewart L, Katial R (2007). Exhaled nitric oxide. Immunol Allergy Clin N Am.

